# Providers’ Perspectives on the Social Determinants of Health and Burnout Among HIV Care Providers in the United States

**DOI:** 10.7759/cureus.102400

**Published:** 2026-01-27

**Authors:** Kristen Khoang, Naima Alam, Alexandra Brown, Matthew Shoemaker

**Affiliations:** 1 Infectious Diseases, University of Kansas School of Medicine, Kansas City, USA; 2 Biostatistics, University of Kansas School of Medicine, Kansas City, USA; 3 Infectious Diseases, University of Kansas Medical Center, Kansas City, USA

**Keywords:** acquired immunodeficiency syndrome (aids), hiv care, human immunodeficiency virus (hiv), provider burnout, social determinants of health (sdoh)

## Abstract

Introduction: Human immunodeficiency virus (HIV) infection can impact the physical health and psychological well-being of those who are infected and requires effective long-term management to achieve viral suppression. A patient’s ability to seek care is largely influenced by social determinants of health (SDOH) and the provider’s ability to help manage social needs.

Objectives: This study aims to elucidate how SDOH impacts the provider’s practice and well-being. We also assessed how provider burnout, which is significantly associated with feeling emotional exhaustion, plays a role in the patient-provider relationship.

Methods: This cross-sectional quantitative study was conducted at the Division of Infectious Diseases of the University of Kansas Medical Center, Kansas City, USA, with 86 providers who care for people living with HIV across the United States who completed an electronic survey about their perceptions of SDOH, barriers to social needs screening, and their personal experience of burnout. Providers identified several unmet social needs, such as financial instability, transportation, and appointment logistics, as major barriers for the patients to engage in HIV care. Data were analyzed using descriptive statistics and chi-square tests to evaluate associations between provider-related variables and provider burnout.

Results: Our results revealed that lack of access to social needs, such as financial instability, transportation, and appointment logistics, and insufficient time were barriers preventing clinicians from inquiring about social needs. We had a total of 86 respondents of healthcare providers: 95% were physicians and 5% were advanced practice practitioners (APPs). Moreover, 35% were located in the Midwest, 28% in the Northeast, 24% in the South, and 13% in the West regions of the United States. Survey respondents indicated that the following SDOH impacted their patients: 60% financial instability, 33% food insecurity, 33% face housing instability, and 49% transportation problems. Sixty-two percent (62%) of the providers reported feeling burned out. Burnout was most frequently reported among providers from the Midwest and the West. Among providers who felt burned out, 26% of those admitted receiving less support from staff and 57% feeling emotional exhaustion. There was a significant association between emotional exhaustion and those who felt burnout. Forty-eight percent (48%) of respondents indicated that they always or often encounter a lack of resources, and 35% identified the lack of time to discuss SDOH as a prominent barrier.

Conclusions: These findings suggest the importance of improving provider well-being and optimizing practices to effectively bridge the community with social services to ensure that people living with HIV remain engaged in care and attain the goal of viral suppression.

## Introduction

Human immunodeficiency virus (HIV) is not only a medical condition that can impact physical health, but it also affects the psychological well-being of people living with HIV (PLWH) due to the social stigma [1]. The infection has a disproportionate impact on certain populations, particularly racial and ethnic minorities, and the lesbian, gay, bisexual, and transgender (LGBT) communities [2]. Geographically, the HIV burden is not evenly distributed by region in the United States. In 2018, the rate of people who received an HIV diagnosis was highest in the South [3]. Even with remarkable medical advancements that could purportedly end the HIV epidemic by 2030, barriers such as stigma and health disparities persist [4,5]. Extensive research has been done to elucidate how the social determinants of health (SDOH), such as poverty, homelessness, food insecurity, and lack of access to transportation, impede patients' ability to engage and be retained in HIV care [6]. Therefore, an integrated approach to address social needs is essential for consistent follow-ups.

One of the central factors that could affect retention in care is the provider-patient relationship. One study revealed that patient satisfaction with the relationship with their providers correlated with better adherence to medication [7,8]. Another study found that the patient-provider relationship positively influenced patients’ appointment attendance [8]. As such, it is vital that HIV providers cultivate mutual understanding, trust, and communication with their patients. Moreover, it is crucial that providers acknowledge unique barriers to healthcare that their patients may experience and consider social needs to make their care delivery more effective. However, many providers reported workload strain, feeling burnt out, and restricted opportunities to develop patient-provider relationships. While they are aware of SDOH, providers have inadequate time and ability to mitigate barriers to health care [9]. Although patients’ perspectives of SDOH and satisfaction with their providers have been studied, evidence regarding providers’ perspectives is limited. Despite substantial research on patient-level SDOH, limited evidence exists on how HIV care providers themselves perceive these challenges and how burnout shapes their clinical practice, making this investigation both timely and necessary. 

The purpose of this study is to assess providers' perception of SDOH, identify barriers to screening for SDOH, and evaluate factors associated with provider burnout.

This article was previously presented as a meeting abstract at the 2024 University of Kansas Medical Center Student Research Forum, held from April 1 to 5, 2024. The abstract was published in the Kansas Journal of Medicine, vol. 17, no. S2 (2024): Supplement 2: Abstracts from the 2024 KUMC Student Research Forum (https://doi.org/10.17161/kjm.vol17.iS2).

## Materials and methods

Participants and setting

This cross-sectional quantitative study was conducted at the Division of Infectious Diseases of the University of Kansas Medical Center, Kansas City, USA. A cross-sectional qualitative study was conducted to assess providers’ perspectives on SDOH that impact patient engagement in HIV care. Survey participants were recruited during the summer and fall of 2023. Survey participants were recruited via targeted emails to academic infectious diseases directors to be shared with their faculty and medical directors of Ryan White clinics to be shared with their providers. In addition, a QR code linked to the survey was randomly distributed among attendees of the annual scientific meeting of the Infectious Diseases Society of America, IDWeek 2023, in Boston, MA. Two groups of survey participants recruited were physicians and advanced practice providers (APPs), who have experience in caring for PLWH. The physician group comprised of attendings and residents in the Infectious Disease specialty and Family Medicine. The APP group was made up of nurse practitioners and physician assistants. Providers practice both in inpatient and outpatient settings. Respondents were grouped based on the location of their practice with the four geographic regions defined by the Centers for Disease Control and Prevention (CDC): West, Midwest, South, and Northeast [10].

Data collection

Participants were asked to complete the survey anonymously with the link provided. No identifiable information was collected. The survey consisted of forty-three questions, and some were modeled after established questionnaires in The Physicians Foundation’s 2022 Survey of America’s Physicians [9] (Table 1).

**Table 1:** Provider survey

Date of consent					
Your role: physician or advanced practice provider					
Which of the following geographic regions do you practice in?	Northeast	Midwest	South	West	
Indicate the extent to which the following barriers prevent your patients from seeking healthcare	A major barrier (5)	Barrier (4)	Neutral (3)	Not a barrier (2)	Not at all a barrier (1)
Appointment logistics (forgetting the appointment, long waiting time, lack of telehealth, etc.)	o	o	o	o	o
Patient’s lack of understanding of the importance of medication and follow-ups	o	o	o	o	o
Cost of office visit and lab tests	o	o	o	o	o
COVID-19 surge	o	o	o	o	o
Social stigma associated with HIV/AID testing and care	o	o	o	o	o
Approximately how many patients do you see a week?					
Approximately what proportion of your patient population do you believe experiences each of the following social determinants of health?					
Financial instability					
Transportation problems					
Housing instability					
Food insecurity					
Respond to the following statements about your use of patients' social needs information:	Always (5)	Often (4)	Sometimes (3)	Rarely (2)	Never (1)
I typically ask patients about their social needs.	o	o	o	o	o
I typically review information regarding members’ social needs from the chart.	o	o	o	o	o
I use information about patients' social needs in medical decisions and care planning.	o	o	o	o	o
To what extent do you agree or disagree with the following statements about your relationships with your patients:	Strongly agree (5)	Agree (4)	Neutral (3)	Disagree (2)	Strongly disagree (1)
You are satisfied with your relationships with your patients.	o	o	o	o	o
The relationships could be improved if you had a longer appointment time for your patients.	o	o	o	o	o
The relationships could be improved if you had a better means to effectively communicate with your patients.	o	o	o	o	o
The relationships could be improved if you had fewer patients you need to see in a day.	o	o	o	o	o
The relationships could be improved if mutual trust were established early.	o	o	o	o	o
Indicate the extent to which the following barriers prevent you from asking patients about their social needs:	A major barrier (5)	Barrier (4)	Neutral (3)	Not a barrier (2)	Not at all a barrier (1)
Lack of time to discuss	o	o	o	o	o
Lack of reimbursement	o	o	o	o	o
Lack of comfort in asking	o	o	o	o	o
Lack of training about how to respond to social needs once they are identified	o	o	o	o	o
Lack of resources to address social needs once they are identified	o	o	o	o	o
To what extent do you agree or disagree with the following statements about incorporating social needs into the care you provide patients:	Strongly agree (5)	Agree (4)	Neutral (3)	Disagree (2)	Strongly disagree (1)
I am aware of the resources available to address patients' social needs.	o	o	o	o	o
I am confident in my ability to help patients address their social needs.	o	o	o	o	o
I am concerned that patients will feel uncomfortable answering questions about their social needs.	o	o	o	o	o
Collecting social needs information is beyond the scope of clinical care.	o	o	o	o	o
I don’t think social needs are an issue for most of my patients.	o	o	o	o	o
Having access to patients' social needs would not change my medical decision-making.	o	o	o	o	o
Please answer Yes or No to the following statements:	Yes	No			
In the last month, did you feel burnout?	o	o			
Do you personally know someone who has lost or been denied a job because of HIV/AIDS?	o	o			
Do you personally know someone who has lost social support because of HIV/AIDS?	o	o			
Do you personally know someone who has lost or been denied education because of HIV/AIDS?	o	o			
Do you personally know someone who has lost or been denied healthcare services because of HIV/AIDS?	o	o			
To what extent do you agree or disagree with the following statements:	Strongly Agree (5)	Agree (4)	Neutral (3)	Disagree (2)	Strongly Disagree (1)
You suffer discrimination or stigma outside of work because you work in the HIV/AIDS-related field.	o	o	o	o	o
You have been experiencing emotional exhaustion and a lack of work-related fulfillment.	o	o	o	o	o
Your decision to work in the HIV field was less supported by your family and friends.	o	o	o	o	o
You receive less support from other staff at the hospital due to the field in which you work.	o	o	o	o	o
You are concerned about your risk of infection while working with infected patients.	o	o	o	o	o
Do you have any additional comments or perspectives you would like to share regarding your patients' adherence to treatment and retention in care?					

The questions were designed to identify the barriers preventing physicians from asking patients about their social needs. Participants rated factors such as lack of time, staffing, training, comfort in asking, and resources. The use of patients' social needs information and how clinicians incorporate social needs into the care they provide was collected. The survey also collected information regarding the SDOH and barriers that may prevent patients’ ability to get care, such as financial instability, transportation problems, housing instability, and food insecurity. Lastly, the quality of the patient-provider relationship was evaluated.

Study data were collected and managed using REDCap electronic data capture tools hosted at the University of Kansas School of Medicine [11,12]. REDCap (Research Electronic Data Capture) is a secure, web-based software platform designed to support data capture for research studies, providing 1) an intuitive interface for validated data capture; 2) audit trails for tracking data manipulation and export procedures; 3) automated export procedures for seamless data downloads to common statistical packages; and 4) procedures for data integration and interoperability with external sources. Access to the REDCap data was restricted to the project personnel.

The Institutional Review Board (IRB) of the University of Kansas Medical Center issued approval (ref. no. STUDY00150258).

Data analysis

After completion of data collection, data from REDCap were exported to an Excel file on a secure server. R Project for Statistical Computing software (version 4.3.0) was used for the analysis. A chi-square test (or Fisher’s exact test where appropriate) was performed to assess if there were any meaningful associations between SDOH variables and providers' perception of HIV patients’ engagement in health care. A chi-square test was utilized to assess relationships between providers' feelings of burnout and factors that might contribute to physician burnout. 

## Results

**Figure 1:**
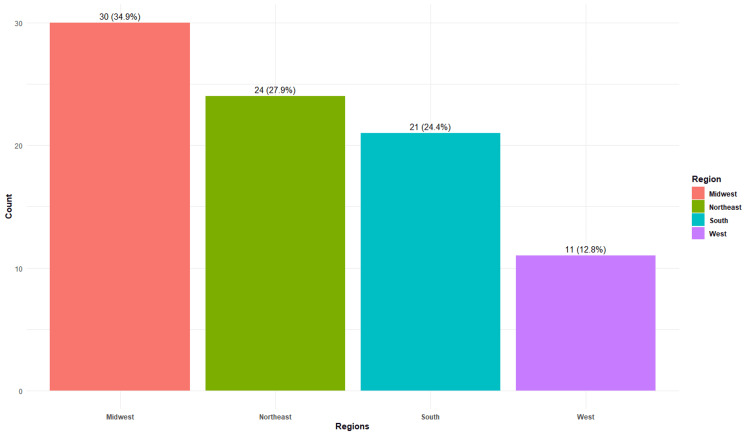
Geographic distribution of the respondants Total N = 86

Among a total of 86 respondents of healthcare providers, 82 (95%) were physicians and four (5%) were APPs, with 30 (35%) located in the Midwest, 24 (28%) in the Northeast, 21 (24%) in the South, and 11 (13%) in the West regions of the United States (Figure 1).

**Figure 2:**
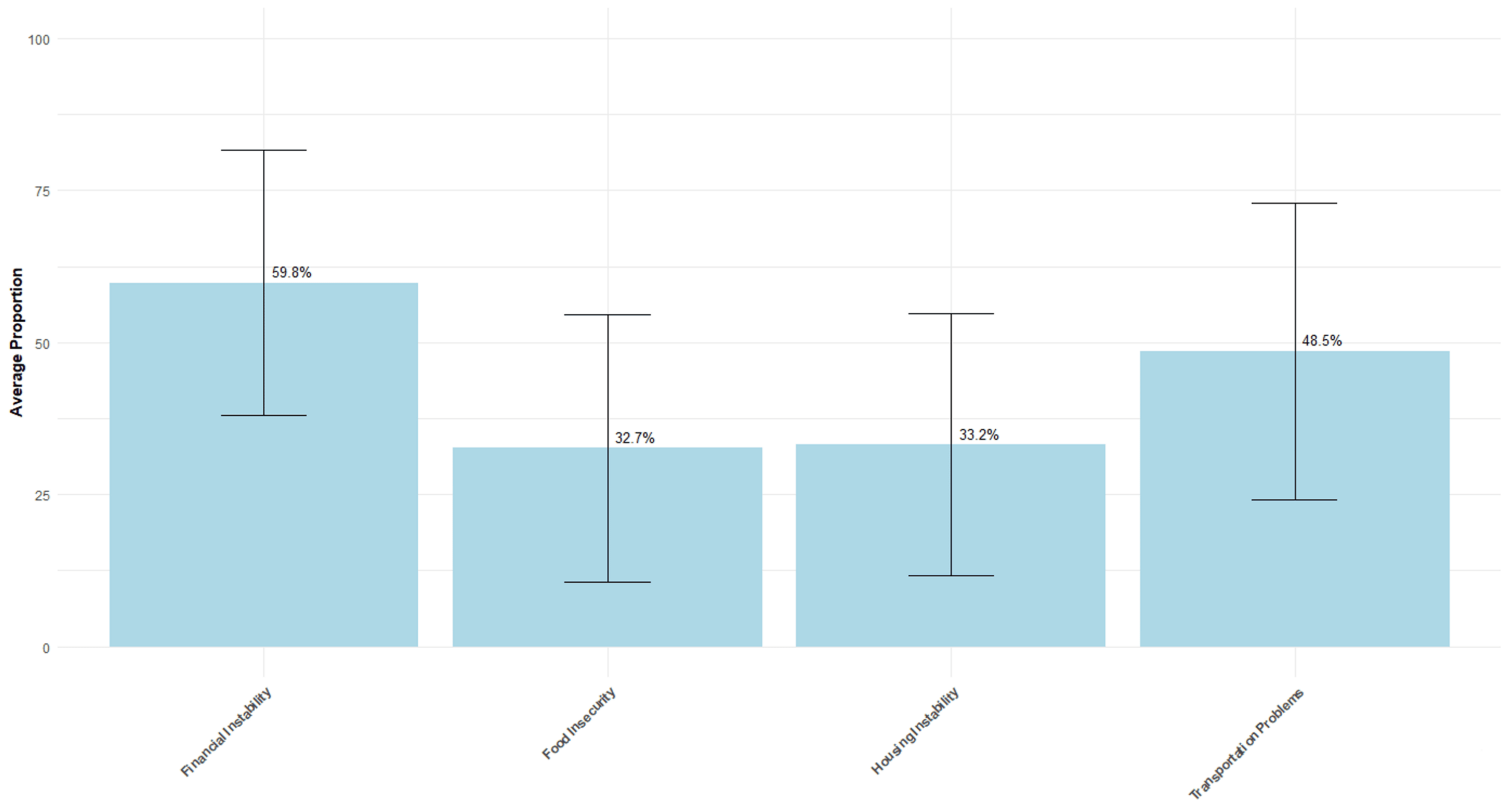
Average proportion of patient populations experiencing issues Total N = 86

When all survey participants were asked about the proportion of patients they see that experience SDOH barriers, they indicated on average that 60% of their overall patient population experience financial instability, 33% experience food insecurity, 33% face housing instability, and 49% have transportation problems (Figure 2).

**Figure 3:**
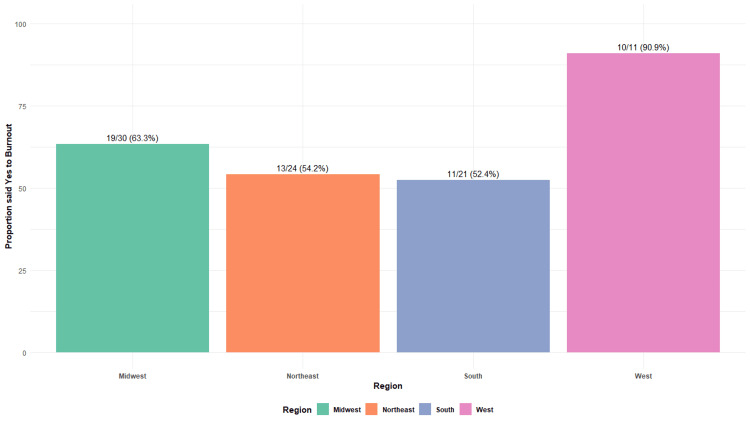
Proportion of providers experiencing burnout by region Total N = 86

Respondents were asked to indicate whether they had experienced burnout in the past month, followed by contributing factors. Overall, 53 (62%) providers reported feeling burned out, while 33 (38%) denied burnout. When examined by region, 30 providers were located in the Midwest, 24 in the Northeast, 21 in the South, and 11 in the West. The proportion of providers reporting burnout in each region was as follows: 19 (63%) in the Midwest, 13 (54%) in the Northeast, 11 (52%) in the South, and 10 (91%) in the West (Figure 3).

**Figure 4:**
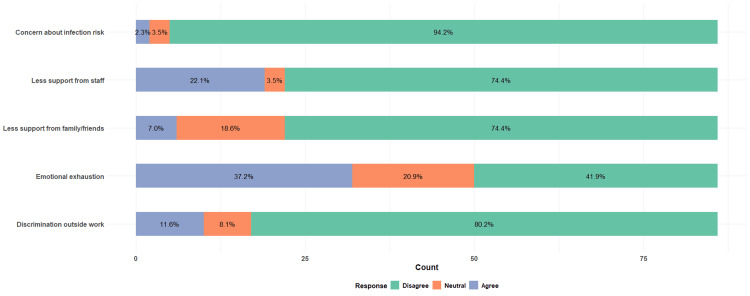
Internal stigma and burnout in all respondents Total N = 86

Out of the total 86 respondents, 80 (94%) disagreed with being concerned about their own risk of infection while working with infected patients, 69 (80%) disagreed with experiencing discrimination outside of work due to the nature of their work in an HIV/AIDS-related field, and 64 (74%) disagreed with having less support from family and friends for their work in the HIV field. Significantly, there was a high proportion of respondents, 19 (22%), who agreed that they received less support from staff due to the field in which they work. Thirty-two (37%) reported experiencing emotional exhaustion and lack of work-related fulfillment (Figure 4).

**Figure 5:**
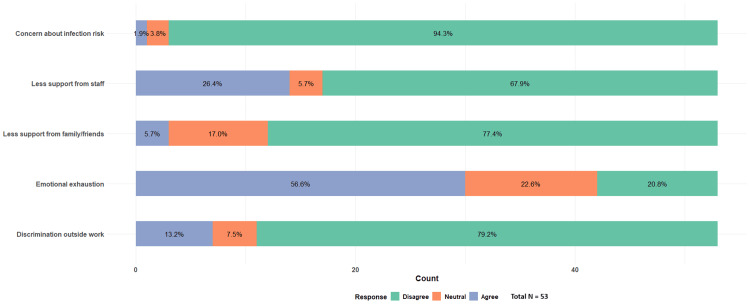
Internal stigma and burnout in providers with burnout Total N = 53

Subsequently, these external factors were analyzed among 53 providers who felt burnout; there was a higher proportion, 14 (26%), who admitted receiving less support from staff, and 30 (57%) felt emotional exhaustion (Figure 5).

The respondents' replies to the survey questions focused on internal stigma and burnout in providers were analyzed using Pearson’s Chi-square test. This analysis revealed a statistically significant association between "emotional exhaustion" and provider burnout, with a p-value less than 0.05. This would suggest that there is a significant association between emotional exhaustion and those who felt burnout at 5% level of significance (Table 2).

**Table 2:** Association check for statements and burnout Total N = 86

	Discrimination outside work	Emotional exhaustion	Less support from family/friends	Less support from staff	Concern about infection risk
Pearson’s Chi-squared test	0.37272	28.854	0.69915	3.8185	0.14691
df	2	2	2	2	2
p-value	0.83	5.426e-07	0.705	0.1482	0.9292
Fisher’s exact test P value	0.8453	1.48e-07	0.6847	0.1937	1

**Figure 6:**
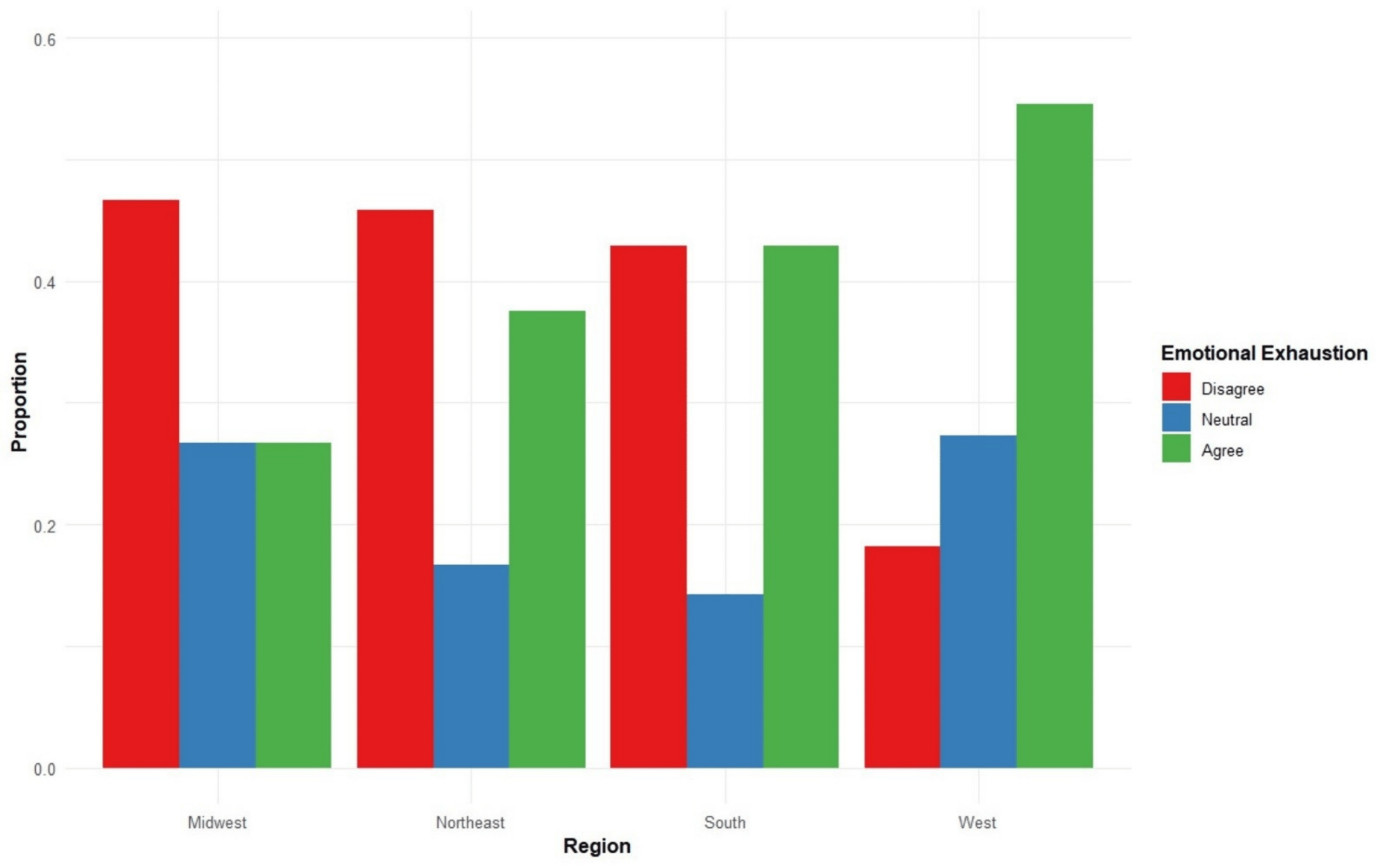
Emotional exhaustion by region Total N = 86

Providers who experienced emotional exhaustion were grouped by the regions in which they practice. A higher proportion of HIV providers in the West region who experienced burnout also experience emotional exhaustion. While there was a high proportion of providers in the Midwest who indicated feeling burned out, fewer reported experiencing emotional exhaustion compared to the other regions (Figure 6).

**Figure 7:**
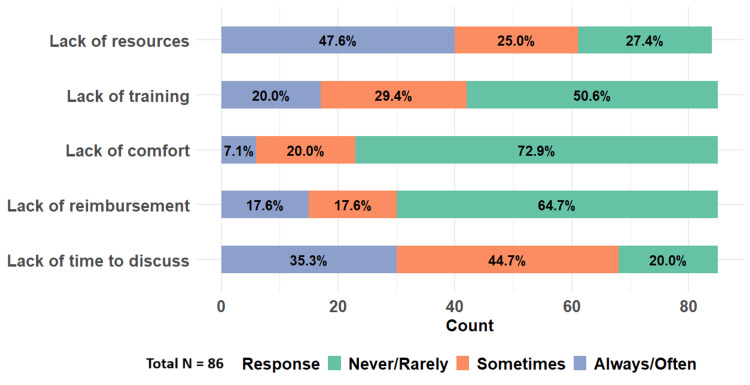
Barriers to social determinants of health (SDOH) screening Total N = 86

In addition, to assess providers’ ability to address SDOH, we asked the participants to identify barriers that prevent them from asking patients about social needs. This includes the lack of time to discuss, reimbursement, comfort in asking and training about how to respond to social needs, and the lack of resources to address social needs once they are identified. As a result, nearly 41 (48%) participants indicated that they always or often encounter a lack of resources, and 30 (35%) identified that the lack of time to discuss was a prominent barrier (Figure 7).

**Figure 8:**
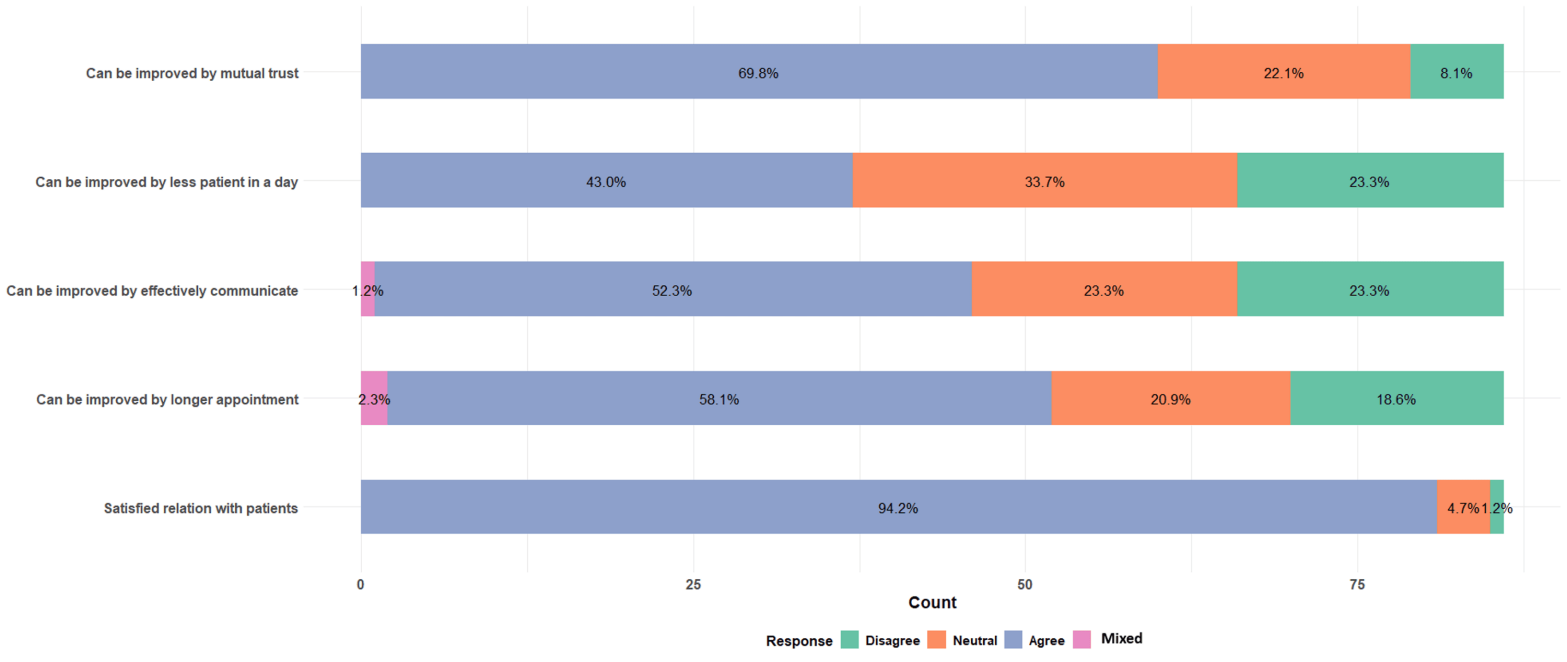
Patient-provider relationship Total N = 86

Lastly, we investigated the patient-provider relationship and identified strategies to foster the relationship (Figure 8).

Providers noted that the relationship could be improved through the early establishment of mutual trust (60 providers (70%)) and better means of effective communication (45 providers (52%)). In addition, many believed that having fewer patients per day (37 providers (43%)) and longer appointment times (50 providers (58%)) would further enrich the provider-patient relationship.

All figures were created by the authors using Microsoft® Excel® for Microsoft 365 MSO (64-bit, version 2508, build 16.0.19127.20484, Microsoft Corp., USA).

## Discussion

SDOH preventing patients from seeking HIV care

SDOH encompasses multiple factors, including limited access to healthcare, education, and economic opportunities, which can negatively affect health outcomes, and the impact is well-documented in the literature. Our study found that providers reported that two-thirds of their HIV patients experience financial instability, half of their patient population struggle with transportation issues, and nearly one-third deal with food insecurity and housing instability. All these factors have a profound impact on patients’ ability to seek care and attend medical appointments. Respondents also indicated medical appointment logistics, such as long wait times and lack of telehealth or transportation, as major barriers. In addition, studies have found that patients have a higher risk of missing appointments for ongoing HIV care if they live in residentially segregated areas and rely on public transportation than if they do not [12,13]. Transportation and the lack of telehealth are ongoing challenges for HIV patients who live in rural communities [14].

As a medical condition associated with socio-economic disparities, HIV infection is considered a pandemic of poverty because it is more prevalent among economically disadvantaged individuals [15]. The CDC reports that HIV prevalence is highest among people who live in poverty, with limited education, and with unstable employment [16]. Like other chronic diseases, socio-economic disparities can have a significant impact on a person's ability to access adequate healthcare. Unlike other chronic diseases, a more integrated, comprehensive approach is required to address health disparities in HIV because of how its transmission intertwines with substance abuse and mental health disorders [17] The optimal care team should consist of primary HIV provider, case management, social support services, adherence counseling, and behavior healthcare [18] This integrated care model would encourage consistent follow-up and help address social barriers that underlie the HIV epidemic. To fulfill our mission of ending the HIV epidemic by 2030, continued effort from the medical community and health policy makers to effectively address SDOH challenges is essential. A respondent from our study remarked on the ongoing challenges, noting that “sometimes the societal barriers are still too great to allow for optimal engagement in health care.”

Barriers to social needs screening

Clinicians are made aware of the influence of SDOH on health outcomes in their training and practice. Addressing SDOH is perceived as a multifaceted, complex task. From our results, we found that the lack of resources and the lack of time are major barriers that prevent HIV clinicians from inquiring about social needs. Other barriers include a lack of training and reimbursement. A quote from a respondent highlighted the barrier, stating, “We know that so many don't have housing or adequate food, but there aren't many resources to connect them with here. It can feel invasive to probe about this when I have nothing to offer.” Our findings are consistent with previously published data regarding implementation barriers once SDOH have been identified [18,19]. Our study suggests that to reduce health disparities, steps should be taken to address these challenges. Perhaps, additional resources should be allocated to the HIV geographic hotspots, regions with more disadvantaged populations, and rural areas. To address the lack of training to help patients with their social needs, continuing medical education or focused training programs could be beneficial to enhance providers' knowledge and efficiency in making resource referrals [20]. Therefore, to encourage effective implementation, social support programs in our healthcare system should consider factors such as time constraints, resource availability, and other structural elements that currently hinder providers from addressing social needs.

Physician burnout

Delivering care in the field of HIV is physically and emotionally demanding because of the complexity of the illness related to a wide range of comorbidities, opportunistic infections, social stigma, and health disparities. An increasing number of people living with HIV (PLWH) also contributes to a higher volume of patients needing care. Thus, HIV providers are more vulnerable to burnout. Our findings contribute to the growing literature regarding the increasing physician burnout rate [21]. We found that nearly two-thirds of providers in the Midwest region experience burnout, and the rate is even more striking in the West region of the US, where over 90% of providers have reported it. As a response to chronic stress, burnout is significantly associated with experiencing emotional exhaustion. There was a higher proportion of providers in the West who experienced both of those conditions. By contrast, fewer burnt-out providers in the Midwest reported feeling emotionally exhausted, suggesting other factors may contribute to burnout in this region. Factors that drive high emotional exhaustion include excessive workload and low work-life balance; consequently, this leads to depletion of mental energy, cynical attitudes, and less empathy toward patients [22,23]. The effects of burnout are particularly detrimental in the field of HIV care, in which long-term management of PLWH and patient-provider relationships are crucial for patients’ retention in care. Our study found that while most providers reported satisfactory relationships with their patients, many indicated the connection with patients could be enhanced by reducing work volume, having longer appointment times, better communication, and mutual trust. Thus, it raises an alarming need for approaches at an individual and organizational level to reduce HIV physician burnout, especially in geographic regions with high HIV prevalence, such as the coastal areas and the Southern part of the US. Some beneficial approaches include developing effective coping skills and establishing a supportive work culture and environment that cultivates professional and personal fulfillment [13]. We also suggest peer support programs where physicians can connect with and receive support from their colleagues, fostering mutual trust and connectedness. Conducting regular well-being check-ins with physicians and having outreach programs facilitating access to counseling or therapy to help physicians cope with the emotional demands of their work would be helpful ways to mitigate burnout.

Limitations

It is worth noting a few limitations to this study. The sample size was smaller than we would have liked. Ideally, we could have elicited more than 100 responses. The small sample size is a result of our recruitment method. Given our recruitment method, these results are subject to sampling bias. In addition, given our recruitment method, we were unable to produce an accurate response rate. As with all survey results, we have to consider how response bias could impact our results. Response bias would likely have a larger impact on these results, given the small sample size. We were unable to compare differences in perspectives of physicians and APPs due to the limited number of APP respondents. We hypothesized there could be a difference in perspectives and burnout levels between the two groups. While it provided enough statistical power for regional comparison, the small sample size of respondents from the West may limit the true representation of this region. Because the identified barriers were from the providers’ point of view, they are attributable to impacting patients’ adherence or retention in health care. Nevertheless, there was no direct measurement of the patient’s retention. Lastly, our statistical evaluation is limited due to the inherent constraints of cross-sectional analyses and an inability to establish causality. 

## Conclusions

We identified several social needs surrounding HIV patients and factors associated with burnout among HIV providers. Our results underscore the interplay between SDOHs, particularly treatable and preventable illnesses, like HIV, and provider burnout. Our findings pave the way for further research, as we have started to outline optimal practices for administering resources in a way that effectively bridges the community with social services in our quest to end the HIV epidemic. These insights may help to guide future policy development aimed at enhancing provider support systems, thus improving the identification of SDOH in HIV care and addressing the structural determinants that shape patient engagement.
